# A Preliminary List of Levels and *g*-Values for the First Spectrum of Thorium (Th I)

**DOI:** 10.6028/jres.063A.022

**Published:** 1959-12-01

**Authors:** Romuald Zalubas

## Abstract

The present state of the analysis of the first spectrum of thorium (Th I) is discussed briefly. Even and odd levels are listed in tables 1 and 2. The low even levels form terms arising from the configurations 6*d^2^ 7s*^2^ and 6*d*^3^ *7s.* The Th I standard wavelengths that fit into the known level arrays are presented in table 3.

## 1. Introduction

Work on thorium spectra was started at the National Bureau of Standards by C. C. Kiess as early as 1926. His data are included in the present work, but have never been published separately.

In 1955 it was decided to obtain a new description and analysis of the Th I spectrum at this Bureau, because electrodeless lamps were available [l].^1^ The description of the thorium spectra will be published soon, and will include an account of the experimental procedures as well as the history of the thorium spectra. Therefore, only the work pertaining to the analysis of Th I will be mentioned here.

In 1946 Ph. Schuurmans [2] published a list of the Th I levels which he had discovered. Five of his even levels (0.00, 2869.18, 5563.15, 6362.39, and 7502.25), and 23 of his odd levels are confirmed by the present analysis. He indicated, also, that 6*d*^2^ *7s*^2 3^F_2_ is the ground state of Th I. His work was based on Zeeman data for 48 Th I lines by Lier [3], and the list of Th I wavelengths by Fred [4].

## 2. Procedure

The new description of thorium spectra contains 16,000 lines between 2000 and 11500 A. About 1,000 Zeeman patterns have been observed and measured, of which 400 are sufficiently well resolved for the determination of *g*-values. The chief difficulty in securing Th I Zeeman data is that the lines are heavily masked by strong Th II patterns. Therefore, two sets of plates had to be measured, one of strong and the other of weak exposures, in order to resolve a greater number of Th I patterns.

In the course of the analysis, Zeeman data from the Massachusetts Institute of Technology became available [5] for about two dozen additional lines. Th I and Th II lines have been separated by using the following criteria: (a) relative intensities in tube and spark exposures, (b) relative intensities of *n* and *p* components of unresolved Zeeman patterns as compared with a no-field exposure, (c) resolved Zeeman patterns, and (d) classified lines of Th II [6], In this way 12,000 lines have been attributed to Th I.

The vacuum wave numbers were calculated on an electronic computer by using Edlén’s formula. The analysis has been substantially speeded up by the use of the computer. Bozman and Coleman [7] have written the codes with which the levels were searched by the computer from known intervals.

## 3. Results

Table 1 contains the even energy levels starting with 6*d*^2^ *7s*^2 3^F_2_ as the ground state zero. The columns of this table read as follows: (1) electron configuration of the term; (2) designation of the term; (3) the inner quantum number or *J*-value of the level; (4) value of the atomic energy level in cm^−1^; (5) *g*-value as observed; (6) theoretical Landé *g*-value in LS-coupling. The electron configurations and term assignments of even levels appear to be fairly definite, and agree well with preliminary theoretical calculations of Trees [8].

Table 2 contains the known odd levels and has the following columns: (1) inner quantum number *J*, (2) value of energy level, and (3) observed *g*-value. Doubtful levels or those that do not have confirmation from Zeeman data are not included in this table.

The *g*-values of even levels are the means from 5 to 40 determinations, those for odd levels have from 1 to 6 determinations. The *g*-values given to three decimal places are derived from 10 or more observations.

By comparison with the ionization potentials of neighboring elements, one can expect the ionization potential of Th I to be between 4 and 5.5 ev. In order to establish levels of configurations 6*p*^2^*7s ns* and 6*d*^3^*ns* with a higher *n*-value, infrared lines beyond the photographic region must be observed.

Only a little more than half of the stronger lines are accounted for at present. The remaining lines, and about 100 well resolved Zeeman patterns indicate that there should exist another system of terms which is probably connected with the known levels by a small number of intercombinations. This idea is supported by the existence of two sets of terms in Th II [6]. The work on the Th I analysis will be continued in this laboratory.

## 4. Secondary Standards

Simultaneously with this work, interferometric measurements of thorium secondary standards have been made by Meggers and Stanley [9]. They have published interferometric wavelengths for 222 thorium lines of which 46 are classified as Th II and 107 are now classified as Th I. Because these lines test the accuracy of the values of energy levels, they are given in table 3 for Th I.

## Figures and Tables

**Table 1:** Even energy levels of *Th I*

Config.	Design	*J*	Level	Obs. *g*	LS. *g*
					
6*d*^2^7*s*^2^	*a* ^3^*F*	2	0.00	0.741	0.667
		3	2869.26	1.074	1.083
		4	4961.66	1.212	1.250
	*a* ^3^P	0	2558.06	0.00	0/0
		1	3865.47	1.477	1.500
		2	3687.99	1.256	1.500
	*a* ^1^D	2	7280.13	1.189	1.000
	*a* ^1^G	4	8111.00	1.08	1.000
6*d*^3^(^4^F)7*s*	a^5^ F	1	5563.14	0.062	0.000
		2	6362.40	1.014	1.000
		3	7502.29	1.253	1.250
6*d*^3^(^4^F)7*s*	*a* ^5^F	4	8800.25	1.310	1.350
		5	9804.81	1.366	1.400
6*d*^3^(^4^P)7*s*	*a* ^5^P	1	11601.03	2.41	2.500
		2	11802.94	1.721	1.833
		3	12847.97	1.39	1.667
6*d*^3^ (^2^G)7*s*	*a* ^3^G	3	13088.57	1.04	0.750
		4	13297.42	0.98	1.050
		5	14204.30	1.13	1.200
6*d*^3^ (^2^D)7*s*	*a* ^3^D	1	13962.50	0.76	0.500
6*d*^3^(^2^H)7*s*	*a* ^3^H	4	15493.23	0.92	0.800

**Table 2:** Odd energy levels of *Th I*

*J*	Level	Obs. *g*
		
2	14032.10	1.15
3	15166.90	1.06
2	16217.48	1.10
3	16671.35	(1.18)
2	17224.30	1.07
1	17354.64	0.51
3	17411.22	1.12
2	17847.10	1.17
4	18053.64	………….
3	18069.10	1.16
0	18382.82	0.00
1	18614.33	1.41
4	18809.92	………….
3	18930.29	0.99
2	19039.15	1.11
3	19503.15	1.10
2	19516.98	1.37
1	19817.17	1.57
4	19948.43	(1.29)
3	20214.93	1.17
1	20423.50	1.42
2	20522.72	0.84
4	20566.69	………….
0	20724.37	0.00
1	20737.28	1.42
2	20922.13	1.16
4	21120.45	(1.03)
3	21165.10	1.31
2	21252.62	0.67
4	21539.59	1.19
1	21668.96	1.56
2	21738.04	(0.53)
3	22141.61	1.10
2	22248.95	1.13
3	22339.00	1.01
1	22396.82	1.54
2	22508.06	1.38
3	22669.90	1.22
3	22855.30	1.09
1	22877.51	0.64
1	23049.46	1.42
2	23093.98	1.32
1	23481.37	0.85
3	23521.06	1.08
2	23603.52	1.39
4	23655.16	(1.20)
1	23741.07	(0.71)
2	23752.67	1.11
2	24182.41	(1.27)
4	24202.57	1.39
2	24307.75	1.51
2	24381.34	1.25
3	24421.08	………….
3	24561.65	1.20
5	24701.06	1.15
3	24769.72	1.15
1	24838.92	0.76
3	24981.10	1.07
3	25321.95	1.35
4	25355.60	0.98
3	25442.69	1.10
1	25526.26	1.08
2	25703.40	1.03
1	25809.30	1.59
4	25877.52	(1.07)
3	26036.36	(0.96)
4	26048.54	1.13
3	26096.98	………….
2	26113.27	0.99
1	26287.05	0.72
2	26363.11	1.02
4	26384.94	(1.07)
3	26508.03	1.08
4	26790.43	1.14
3	26878.16	0.88
3	26995.78	1.18
2	27061.40	(0.94)
1	27087.99	(1.14)
3	27260.17	1.12
4	27266.03	1.12
3	27317.39	(1.07)
3	27670.95	1.26
2	27674.33	1.09
2	27784.37	0.85
5	27852.75	1.26
4	27948.61	1.28
1	28024.69	1.03
2	28347.55	(1.55)
1	28372.69	1.79
2	28513.32	(1.10)
3	28589.29	1.17
1	28649.15	1.11
3	28676.29	1.02
3	28884.97	1.17
2	28917.96	0.95
4	28932.65	1.09
5	29050.77	1.14
1	29157.10	0.89
3	29157.88	1.17
1	29197.33	1.16
2	29252.82	1.00
2	29419.25	1.78
1	29640.28	0.98
3	29686.37	1.27
3	29744.52	1.06
2	29853.14	0.92
3	30017.10	1.15
3	30255.45	1.12
1	30281.04	(1.48)
4	30517.42	1.46
2	30553.29	1.05
1	30723.82	1.07
3	30761.72	1.21
2	30813.00	0.93
1	30928.73	0.99
3	30990.52	1.07
3	31283.12	1.13
3	31523.96	1.11
2	31599.36	1.18
1	31712.73	1.16
3	31780.87	1.17
2	31870.08	0.93
4	31953.46	1.06
1	32080.39	0.80
3	32197.12	1.13
3	32285.23	(1.09)
4	32439.05	(1.20)
2	32575.41	0.78
1	32665.59	(0.83)
4	32862.51	(1.16)
3	33043.35	1.15
1	33161.80	(1.32)
4	33270.59	(1.16)
2	33297.13	(1.23)
3	33591.20	1.00
3	33800.68	1.16
5	33844.96	1.10
4	33956.93	………….
4	34001.33	1.04
2	34371.82	(1.32)
1	34590.97	(1.48)
3	34704.42	(1.13)
5	35081.03	(1.08)
4	35131.22	1.26
5	35273.95	1.18
4	35351.44	(1.27)
2	35533.34	0.83
4	36062.87	1.11
2	36189.01	(1.98)
5	36275.19	1.00
1	36361.49	1.11
4	36382.66	(1.07)
5	36837.96	1.18
3	36871.99	………….
5	37008.75	1.12
2	37149.18	1.05
4	37605.80	1.09
3	38216.95	………….
3	39611.56	(1.25)

**Table 3:** Classified standards of *Th I*

Wavelength in air	Relative intensity	Classification
		
*A*		
6943.6112	600	*a* ^5^F_5_— $2420_{4}^{{}^{\circ}}$
6829.0355	150	*a* ^5^F_3_— $2214_{3}^{{}^{\circ}}$
6756.4528	250	*a* ^3^P_0_— $1735_{1}^{{}^{\circ}}$
6727.4585	200	*a* ^5^F_1_— $2042_{1}^{{}^{\circ}}$
6678.7076	30	*a* ^1^D_2_— $2224_{2}^{{}^{\circ}}$
6662.2694	250	*a* ^5^F_3_— $2250_{2}^{{}^{\circ}}$
6591.4849	100	*a* ^3^F_2_— $1516_{3}^{{}^{\circ}}$
6588.5398	200	*a* ^3^P_1_— $1903_{2}^{{}^{\circ}}$
6554.1605	100	*a* ^3^F_4_— $2021_{4}^{{}^{\circ}}$
6531.3423	400	*a* ^5^F_2_— $2166_{1}^{{}^{\circ}}$
6490.7378	120	*a* ^5^F_4_ — $2420_{4}^{{}^{\circ}}$
6413.6152	200	*a* ^3^G_4_— $2888_{3}^{{}^{\circ}}$
6411.8996	250	*a* ^5^F_3_— $2309_{2}^{{}^{\circ}}$
6342.8600	300	*a* ^5^F_4_— $2456_{3}^{{}^{\circ}}$
6257.4237	100	*a* ^5^F_2_— $2233_{3}^{{}^{\circ}}$
6224.5275	100	*a* ^3^F_3_— $1893_{3}^{{}^{\circ}}$
6207.2205	160	*a* ^5^F_1_— $2166_{1}^{{}^{\circ}}$
6191.9054	100	*a* ^5^F_2_— $2250_{2}^{{}^{\circ}}$
6182.6219	400	*a* ^3^F_3_— $1903_{2}^{{}^{\circ}}$
6151.9932	120	*a* ^5^F_3_— $2375_{2}^{{}^{\circ}}$
6049.0510	100	*a* ^3^P_2_— $2021_{3}^{{}^{\circ}}$
6037.6978	140	*a* ^3^P_1_— $2042_{1}^{{}^{\circ}}$
6007.0725	180	*a* ^5^F_4_— $2544_{3}^{{}^{\circ}}$
5975.0656	250	*a* ^5^F_2_— $2309_{2}^{{}^{\circ}}$
5973.6651	250	*a* ^3^P_2_— $2042_{1}^{{}^{\circ}}$
5938.8255	140	*a* ^5^F_1_— $2239_{1}^{{}^{\circ}}$
5885.7017	120	*a* ^5^F_5_— $2679_{4}^{{}^{\circ}}$
5804.1414	300	*a* ^3^F_2_— $1722_{2}^{{}^{\circ}}$
5789.6439	200	*a* ^5^F_3_— $2476_{3}^{{}^{\circ}}$
5760.5510	600	*a* ^3^F_2_— $1735_{1}^{{}^{\circ}}$
5725.3887	250	*a* ^5^F_5_— $2726_{4}^{{}^{\circ}}$
5615.3202	350	*a* ^3^P_1_— $2166_{1}^{{}^{\circ}}$
5587.0265	500	*a* ^3^F_4_— $2285_{3}^{{}^{\circ}}$
5579.3585	300	*a* ^5^F_1_— $2348_{1}^{{}^{\circ}}$
5573.3538	350	*a* ^1^G_4_— $2604_{4}^{{}^{\circ}}$
5558.3426	400	*a* ^1^G_4_— $2609_{3}^{{}^{\circ}}$
5548.1761	300	*a* ^5^F_2_— $2438_{2}^{{}^{\circ}}$
5539.2615	400	*a* ^5^F_5_— $2785_{5}^{{}^{\circ}}$
5509.9937	300	*a* ^5^F_5_— $2794_{4}^{{}^{\circ}}$
5499.2552	250	*a* ^3^P_0_— $2073_{1}^{{}^{\circ}}$
5431.1116	300	*a* ^5^F_2_— $2476_{3}^{{}^{\circ}}$
5417.4856	200	*a* ^3^P_2_— $2214_{3}^{{}^{\circ}}$
5386.6109	300	*a* ^3^F_4_— $2352_{3}^{{}^{\circ}}$
5343.5813	500	*a* ^3^P_2_— $2239_{1}^{{}^{\circ}}$
5326.9755	400	*a* ^1^G_4_— $2687_{3}^{{}^{\circ}}$
5258.3609	300	*a* ^3^P_1_*—* $2287_{1}^{{}^{\circ}}$
5231.1596	900	*a* ^3^P_0_— $2166_{1}^{{}^{\circ}}$
5158.6041	700	*a* ^3^F_3_— $2224_{2}^{{}^{\circ}}$
5002.0968	400	*a* ^3^F_3_— $2285_{3}^{{}^{\circ}}$
4894.9546	350	*a* ^3^F_2_— $2042_{1}^{{}^{\circ}}$
4878.733	200	*a* ^3^P_0_— $2304_{1}^{{}^{\circ}}$
4865.4769	350	*a* ^3^G_4_— $3384_{5}^{{}^{\circ}}$
4840.8426	400	*a* ^3^F_3_— $2352_{3}^{{}^{\circ}}$
4789.3867	300	*a* ^3^P_2_— $2456_{3}^{{}^{\circ}}$
4766.6001	200	*a* ^3^P_1_— $2483_{1}^{{}^{\circ}}$
4703.9897	500	*a* ^3^F_2_— $2125_{2}^{{}^{\circ}}$
4686.1944	1200	*a* ^3^F_3_— $2420_{4}^{{}^{\circ}}$
4668.1720	700	*a* ^5^F_3_— $2891_{2}^{{}^{\circ}}$
4663.2021	200	*a* ^3^F_3_— $2430_{2}^{{}^{\circ}}$
4595.4198	600	*a* ^3^P_2_— $2544_{3}^{{}^{\circ}}$
4555.813	500	*a* ^3^P_1_— $2580_{1}^{{}^{\circ}}$
4493.3335	1200	*a* ^3^F_2_— $2224_{2}^{{}^{\circ}}$
4482.1694	300	*a* ^3^F_4_— $2726_{4}^{{}^{\circ}}$
4458.0018	600	*a* ^3^P_2_— $2611_{2}^{{}^{\circ}}$
4408.8828	600	*a* ^3^P_2_— $2636_{2}^{{}^{\circ}}$
4378.1768	500	*a* ^3^F_3_— $2570_{2}^{{}^{\circ}}$
4374.1244	600	*a* ^3^F_2_— $2285_{3}^{{}^{\circ}}$
4315.2544	400	*a* ^3^F_3_— $2603_{3}^{{}^{\circ}}$
4257.4959	700	*a* ^3^F_2_— $2348_{1}^{{}^{\circ}}$
4235.4635	600	*a* ^3^F_2_— $2360_{2}^{{}^{\circ}}$
4208.8907	3000	*a* ^3^F_2_— $2375_{2}^{{}^{\circ}}$
4193.0165	900	*a* ^1^G_4_— $3195_{4}^{{}^{\circ}}$
4158.5351	800	*a* ^5^F_5_— $3384_{5}^{{}^{\circ}}$
4115.7587	800	*a* ^5^F_1_— $2985_{2}^{{}^{\circ}}$
4100.3412	1100	*a* ^3^F_2_— $2438_{2}^{{}^{\circ}}$
4067.4507	400	*a* ^3^F_3_— $2744_{2}^{{}^{\circ}}$
4059.2525	1000	*a* ^5^F_2_— $3099_{3}^{{}^{\circ}}$
4043.3945	800	*a* ^3^F_4_— $2968_{3}^{{}^{\circ}}$
4036.0475	1800	*a* ^3^F_2_— $2476_{3}^{{}^{\circ}}$
4012.4950	2000	*a* ^3^F_3_— $2778_{2}^{{}^{\circ}}$
3923.7993	400	*a* ^3^F_3_— $2834_{2}^{{}^{\circ}}$
3869.6635	600	*a* ^5^F_2_— $3219_{3}^{{}^{\circ}}$
3839.6941	2500	*a* ^3^F_2_— $2603_{3}^{{}^{\circ}}$
3828.3845	3200	*a* ^3^F_2_— $2611_{2}^{{}^{\circ}}$
3803.0750	4000	*a* ^3^F_2_— $2628_{1}^{{}^{\circ}}$
3771.3703	1500	*a* ^3^F_2_— $2650_{3}^{{}^{\circ}}$
3762.9345	1200	*a* ^3^P_2_— $3025_{3}^{{}^{\circ}}$
3727.9022	800	*a* ^3^F_3_— $2968_{3}^{{}^{\circ}}$
3719.4345	3000	*a* ^3^F_2_— $2687_{3}^{{}^{\circ}}$
3700.9780	300	*a* ^5^F_1_— $3257_{2}^{{}^{\circ}}$
3692.5661	1200	*a* ^3^P_2_— $3076_{3}^{{}^{\circ}}$
3682.4861	1000	*a* ^3^F_3_— $3001_{3}^{{}^{\circ}}$
3669.9687	750	*a* ^1^G_4_— $3535_{4}^{{}^{\circ}}$
3656.6936	1000	*a* ^3^F_3_— $3020_{2}^{{}^{\circ}}$
3642.2487	2200	*a* ^3^F_2_— $2744_{2}^{{}^{\circ}}$
3622.7951	800	*a* ^3^P_2_— $3128_{3}^{{}^{\circ}}$
3612.4271	1400	*a* ^3^F_2_— $2767_{2}^{{}^{\circ}}$
3598.1196	2000	*a* ^3^F_2_— $2778_{2}^{{}^{\circ}}$
3584.1753	800	*a* ^3^F_3_— $3076_{3}^{{}^{\circ}}$
3576.5573	1000	*a* ^1^G_4_— $3606_{4}^{{}^{\circ}}$
3567.2635	1200	*a* ^3^F_2_— $2802_{1}^{{}^{\circ}}$
3544.0176	1500	*a* ^5^F_4_— $3700_{5}^{{}^{\circ}}$
3518.4033	1000	*a* ^3^F_3_— $3128_{3}^{{}^{\circ}}$
3451.7019	900	*a* ^3^P_2_— $3265_{1}^{{}^{\circ}}$
3442.5785	800	*a* ^3^F_4_— $3400_{4}^{{}^{\circ}}$
3405.5575	1400	*a* ^3^P_2_— $3304_{3}^{{}^{\circ}}$
3396.7273	1400	*a* ^3^P_1_— $3329_{2}^{{}^{\circ}}$
3380.8595	900	*a* ^3^F_3_— $3243_{4}^{{}^{\circ}}$
3330.4765	1800	*a* ^3^F_2_— $3001_{3}^{{}^{\circ}}$
3309.3650	800	*a* ^3^F_2_— $3020_{2}^{{}^{\circ}}$
3304.2381	3000	*a* ^3^F_2_— $3025_{3}^{{}^{\circ}}$

## Footnotes

1 Figures in brackets indicate the literature references at the end of this paper.
